# Nutraceutical Combinational Therapy for Diarrhoea Control with Probiotic Beverages from Fermented Fruits, Vegetables and Cereals to Regain Lost Hydration, Nutrition and Gut Microbiota

**DOI:** 10.3390/microorganisms11092190

**Published:** 2023-08-30

**Authors:** Divakar Dahiya, Poonam Singh Nigam

**Affiliations:** 1 Wexham Park Hospital, Wexham Street, Slough SL2 4HL, UK; 2 Biomedical Sciences Research Institute, Ulster University, Coleraine BT52 1SA, UK

**Keywords:** beverage, cereals, diarrhoea, fermentation, fruits, gut, microbiota, nutraceutical, nutrition, probiotic, vegetables

## Abstract

This article deals with the condition of irregular bowel movements known as diarrhoea, its pathology, symptoms and aetiology. The information has been presented on causes of diarrhoea that include gut infections, food intolerances and allergies to certain ingredients, problems in the gastrointestinal tract like irritable bowel syndrome, inflammatory bowel disease and, the condition of dysbiosis which occurs due to long-term use of antibiotics, or other medicines, etc. Most cases of diarrhoea can be resolved without needing medical treatment; however, it is still important to avoid dehydration of the body and use some supplements to get necessary nutrients which are lost with frequent bowel movements before they can get absorbed and assimilated in the gastrointestinal tract. Probiotic products are reported as natural therapeutic agents, which can reduce the risk of diarrhoea in both adults and children. The intake of dietary fluid supplements in the form of fermented beverages containing probiotic strains could help in diarrhoea control. The patient would achieve benefits with the consumption of these functional beverages in three ways—by regaining lost fluids to the body, supplementing beneficial gut bacteria to restore diversity in gut microbiota, which was disturbed in the condition of diarrhoea as well as regaining a source of quick nutrition to recoup energy.

## 1. Introduction

Our digestive system is remarkably efficient in regulating food digestion, assimilation of nutrients and the excretion process. After the digestion of food and absorption of nutrients, it gets rid of all the waste material from the body through regular bowel movements. Healthy bowel movements usually empty the intestine of undigested food, harmful bacteria and their toxins. However, some people might suffer, for some reason, from disorders in the intestine’s normal functioning, causing an irregular and frequent bowel movement like diarrhoea. The pathology of diarrhoea describes it as the frequent passing of loose watery stool, which results in deficiencies in dietary nutrients and loss of water from the body [1]. Most cases of diarrhoea can be resolved without needing medical treatment. However, severe conditions of diarrhoea can happen where more than 10 watery bowel movements happen a day or frequent diarrhoea where the loss of fluid from the body significantly exceeds the oral intake. Such conditions would cause dehydration in a person, which could be life-threatening if it is not treated in time.

A clear definition and facts about diarrhoea have been provided by the US Department of Health and Human Services, National Institute of Diabetes and Digestive and Kidney Diseases. This service translates and disseminates research findings to increase knowledge and understanding about health and disease among patients, health professionals and the general public [2]. The Centers for Disease Control and Prevention have mentioned in their document, Diarrhoea: Common Illness, Global Killer, that diarrhoea kills 2195 children every day—more than AIDS, malaria and measles combined. However, most diarrhoeal deaths could be preventable using simple, low-cost fluid and dietary interventions [3]. The aim of this article is to review the causes, symptoms and pathology of diarrhoea. For the control of non-acute diarrhoea, a nutraceutical therapy could be taken by patients through the consumption of nutraceutical probiotic beverages. Although in acute conditions, patients need medical treatment; however, combinational therapy with nutraceuticals could also be useful. This article has presented information on some nutraceutical beverages, which could be prepared from the fermentation of fruits, vegetables and cereals by specific strains of probiotic cultures. Such probiotic health drinks would help patients regain lost hydration and nutrition and maintain the disturbed gut microbiota.

## 2. Pathology of Diarrhoea

Based on pathological conditions experienced by the patient, diarrhoea can be classified as below [4]

### 2.1. Acute Diarrhoea

The most common type is acute diarrhoea, where a person suffers from loose watery diarrhoea that lasts for a shorter period of one to two days. This type is a temporary condition of diarrhoea that does not need any treatment and it usually clears itself away after a few days.

### 2.2. Persistent Diarrhoea

This type of diarrhoea generally continues for a longer time of several weeks, for example, from two to four weeks.

### 2.3. Chronic Diarrhoea

In rare cases, diarrhoea starts and stops regularly over a longer period of time. If it lasts for more than four weeks, this pathology is referred to as chronic diarrhoea [5]. Most diarrhoea clears away on its own within four days and finding the cause is not necessary.

## 3. Causes of Diarrhoea

There could be different causes for the onset of diarrhoea in different people. Some common causes observed among populations, regardless of their geographical habitat, include gastric infections, allergies and intolerances to certain foods or preservatives and chemical additives in food and beverages [6], digestive tract problems, abdominal surgery and long-term use of medicines can cause chronic diarrhoea. Diarrhoea can be caused due to some infections from bacteria and parasites that do not go away without treatment. Nonetheless, the colon, where the excretion material is formed, reacts to something abnormal in the colon and can cause its irregular functioning. The most common causes of acute and persistent diarrhoea are infections, travellers’ diarrhoea and side effects of medicines. Medicines that may cause diarrhoea include antibiotics, antacids containing magnesium and medicines used to treat cancer.

### 3.1. Gut Infections Causing Diarrhoea

A bowel infection named gastroenteritis is a common cause in both adults and children. Infections in the digestive tract that spread through foods or drinks are called foodborne illnesses. Gastric infections lasting more than two weeks and less than four weeks can cause persistent diarrhoea. The types of gastroenteritis infections that can cause diarrhoea include:

#### 3.1.1. Viral Infections

An intestinal infection known as viral gastroenteritis causes watery diarrhoea, stomach cramps and occasionally patients experience fever with the feeling of nausea or vomiting. Viral gastroenteritis is also called stomach flu by general people. It can develop through contact with an infected patient and by the use of contaminated food or water. Many viruses cause diarrhoea, including norovirus and rotavirus. Viral gastroenteritis is a common cause of acute diarrhoea [1,2,3,4]; (https://www.mayoclinic.org/diseases-conditions/viral-gastroenteritis/symptoms-causes/syc-20378847 accessed on 8 August 2023).

#### 3.1.2. Bacterial Infections

Several types of bacteria can enter the human body through the intake of contaminated food causing food poisoning, which starts irregular bowel movement. Common bacteria that cause diarrhoea include *Campylobacter*, *Escherichia coli* (*E. coli*)*, Salmonella* and *Shigella.*

#### 3.1.3. Parasitic Infections

Parasites normally enter the human body through the use of contaminated water for washing, bathing or drinking. In due course of time, these parasites flourish and settle down in the digestive tract disturbing the balance and activities of microbiota which normally resides in the gastrointestinal tract. Known parasites that cause diarrhoea include *Cryptosporidium enteritis*, *Entamoeba histolytica* and *Giardia lamblia*.

#### 3.1.4. Travellers’ Diarrhoea

Some people while travelling from one to another country are exposed to different conditions of the environment, climate and local provisions of dietary sources. Travellers’ diarrhoea in most cases is caused by eating food or drinking water, which could be contaminated with harmful bacteria, viruses or parasites. It is most often acute and can be a problem for people travelling to those areas with the problem being in the supply of clean water or unhygienic environmental conditions [7]. However, some parasites ingested through the consumption of dirty water which is not safe for use can cause diarrhoea that could last longer. More information is available on Traveller’s Health, Travellers’ Diarrhoea in a Document by the Centers for Disease Control and Prevention [8].

### 3.2. Diarrhoea Due to Food Intolerances and Allergies to Certain Ingredients Used in Food

After having a gastric infection, people may have problems digesting carbohydrates such as lactose or proteins in foods such as cow’s milk, and products prepared with milk or soy. A person having problems with the digestion of carbohydrates or proteins might get prolonged diarrhoea [9]. Lactose intolerance is a common condition that may cause diarrhoea after eating foods or drinking liquids that contain milk or milk products [10], therefore, research has been focused on the development of dairy-free food and beverage products based on plant-sourced materials [11,12].

Application of sweeteners in some low-calorie or zero-calorie food items, snacks and beverages, some of which could be nutritive [13] or non-nutritive in property [14], is very common in food and drink industries. Fructose is added to many foods and soft drinks as a sweetener called high-fructose corn syrup. In some consumers, fructose intolerance is a condition that may cause diarrhoea after eating foods or drinking beverages that contain fructose as a sweetener. Alternative sweeteners used to replace traditional table sugar sucrose [15] are specific sugar alcohols for example sorbitol, mannitol and xylitol [16,17]. The most used confectionery products are candies and chewing gums, which often include these sugar alcohols, which might cause diarrhoea only in some people. Even though the consumption of sugar-free products prepared with mixed sweeteners [18] could be the occasional cause.

### 3.3. Diarrhoea Due to Problems in the Gastrointestinal Tract

Irritable bowel syndrome (IBS) affects the normal functioning of the large intestine and patients suffer from abdominal pain and altered bowel habits like constipation and diarrhoea. Some people have chronic bowel diseases that could cause chronic diarrhoea, which can be a symptom of chronic IBS and Inflammatory Bowel Disease (IBD) [19]. Digestive tract problems that may cause chronic diarrhoea include celiac disease, Crohn’s disease and other gastrointestinal tract (GIT) disorders like small intestinal bacterial overgrowth, ulcerative colitis, etc. This has been noted in some patients that had abdominal surgery also developed chronic diarrhoea after the surgery, which was performed in a part of GIT like the small intestine, colon or appendix [20].

The aetiology of diarrhoea has been described in Merck Manual Professional Version: Diarrhoea. Normally, 99% of the fluid is absorbed in the small intestine and colon, which results from oral intake of fluid from different items (beverages and food) and secretions from the gastrointestinal tract. That counts to a total daily fluid load of about 9 of 10 L. Consequently, even small reductions of 1% in intestinal water absorption or increases in GIT secretion can increase water content, which is enough to change the stool consistency and might cause diarrhoea. Several basic mechanisms cause the most clinically significant diarrhoea. The three most common mechanisms described in its aetiology are increased osmotic load, increased secretions/decreased absorption and decreased contact time/surface area. In many disorders, more than one mechanism could be active. For instance, a person with inflammatory bowel disease might suffer from diarrhoea due to several mechanisms, such as mucosal inflammation, exudation into the lumen and something that brings secretions and bacterial toxins that affect the function of enterocytes [21].

### 3.4. Diarrhoea Caused Due to Dysbiosis

Long-term use of medicines may cause chronic diarrhoea. Specific treatments using different classes of chemical antibiotics can change the normal gut flora and produce the condition of dysbiosis. Very often antibiotics can cause diarrhoea because these strong chemical drugs kill both the infectious pathogen as well as the gut-friendly bacteria. Under such circumstances, a loss in gut microbiota could result in a condition of diarrhoea during or after antibiotic treatment of other diseases. Gut dysbiosis increases the chances of infection, e.g., with *Clostridioides* (earlier classification *Clostridium difficile*), a bacterium that can cause chronic diarrhoea. Chemotherapy-induced diarrhoea is a common adverse side effect of chemicals used for treatment in cancer patients, which, if not treated, may lead to the discontinuation of the drug being used for a cancer cure and hence the treatment of the patient leads to a failure. Some probiotics such as *Lactobacillus*, *Bifidobacterium* and *Saccharomyces* species have been gaining clinical attention in alleviating chemotherapy-induced adverse events including diarrhoea [22].

## 4. Probiotics as Natural Therapeutic Agents

Probiotics can reduce the risk of diarrhoea in both adults and children [23]. Probiotics are beneficial bacteria that have long been studied to ease digestive problems and improve bowel movements [24]. Probiotics used with prebiotics in synbiotic preparations have appeared as potential therapeutic strategies to restore and develop intestinal microbiota, offering promising solutions for improving bowel function [25]. Adequate administration of live microorganisms called probiotics results in positive effects on health. They can include strains of *Lactobacillus, Bifidobacterium* and other beneficial bacteria, which exercise their effects by modulating the composition of gut microbiota and promoting the healthy functioning of intestinal ecology [26]. A report by O’Toole et al. described the expanding spectrum of probiotics to live biotherapeutics [27]. An industrially important research study conducted by Bosnea et al. reported that the cell viability of probiotic cultures can be enhanced for their application in products requiring live cells with probiotic activity sustained for a longer period of time. For this purpose, researchers used biopolymer-based coacervate structures for the growth adaptation of probiotic bacterial cells [28].

Probiotics can also help with diarrhoea control by suppressing the pathogenic microorganisms that cause diarrhoea and helping the body fight them. The effect of probiotic intake was investigated in the prevention of acute diarrhoea in children residing in an urban slum, where studies were conducted in a community-based, randomised and double-blind placebo-controlled field trial. Research conducted by research team at the National Institute of Cholera and Enteric Diseases (NICED) concluded that when a probiotic fermented milk drink containing *Lactobacillus casei* strain Shirota was consumed by children in the age group of 1–5 years, for 12 weeks, the incidence of diarrhoea reduced significantly by 14% [29]. Collinson and his colleagues conducted another study whether probiotics help to treat acute infectious diarrhoea. In cases of acute infectious diarrhoea, probiotics may act against the harmful microbes that could be the main cause of diarrhoea [30]. Probiotic strains have been reported to help the gut in evading pathogens or reduce inflammation and damage that happens to the gut [19]. A study at McMaster University showed a link between a probiotic and mood improvement in adults suffering from Irritable Bowel Syndrome. The patients were less depressed when they consumed a probiotic. The clinical potential of probiotic microbial strains, used in fermentation for food, beverages and in synbiotic supplements, has been identified through Gut–Brain Signalling [31]. Probiotic strains have applications in the biocatalysis process for the production of health-promoting compounds like GABA for pharmaceutical industries [32].

The time taken for a probiotic to act will depend on several factors including the probiotic strain, product quality, dose and health condition. Researchers are still trying to understand the various benefits of probiotics and it appears that they keep the digestive system in balance, improve digestion of lactose, improve digestive disorders like bloating, pain, constipation and diarrhoea and produce certain vitamins [33]. Because many microbes used as probiotics already exist in our bodies and there are decades of their safe use in functional foods in almost every country, probiotic foods are considered safe for their consumption for providing health benefits [34]. They have no side effects and can be easily incorporated as a part of the daily diet to ensure regular bowel movements and a healthy intestine [35]. Zhao et al. conducted a prospective randomised and controlled trial that reported the effects of fibre and probiotics on diarrhoea associated with enteral nutrition in gastric cancer patients [36]. The therapeutic effect of probiotics on the restoration of bowel function was studied by Yoon et al. in a Randomised Controlled Trial and in rectal cancer patients after their ileostomy closure [37]. Barclay et al. suggested the role of probiotics in nausea, vomiting and return of bowel function after colorectal surgery [38]. Probiotics and prebiotics have been recommended for restoring intestinal health after disease in clinical applications [39].

### Specific Strains of Probiotic Used in Fermentation for Nutraceutical Beverages

The use of Probiotics has been suggested for the Regulation of Health [40]. Pure viable cultures of bacteria and yeasts identified and characterised as probiotic strains are used in the fermentation process to prepare functional beverages. Probiotics used in fermentation usually comprise bacteria, mainly *Lactobacillus*, *Bacillus*, *Bifidobacterium, Streptococcus* and *Enterococcus*, although some strains of the yeast *Saccharomyces* genera have also been included in the list of probiotic cultures. The fermented beverages are normally dairy-based and prepared using milk as the starting raw material. For vegans and lactose-intolerant consumers, various other alternative materials, such as fruits, vegetables, grains, nuts, etc. can be used as suitable substrates to perform the fermentation process. The fermented products prepared with such dietary materials can be consumed as a source of nutrition and are also rich in active probiotics necessary for the maintenance of gut microbiota [6,10,11,12]. *Lactobacillus rhamnosus* GG, *Saccharomyces boulardii*, *Bifidobacterium lactis* and *Lactobacillus casei* are some of the most effective strains of probiotics for treating diarrhoea. Without treatment, about 34 out of 100 people who did not take probiotics were diarrhoea free after three days. With treatment, about 55 out of 100 people who took probiotics were diarrhoea free after three days. Consumption of probiotics helps in restoring the intestinal barrier protecting it from infective strains which could cause gut infection and diarrhoea [41]. Recent research reports probiotics can be used as supplements in the prevention and treatment of infectious diseases [42]. A network meta-analysis conducted by Ioannidis et al. reported the efficacy of probiotics, prebiotics and synbiotics in patients who have undergone an abdominal operation, in terms of bowel function post-operatively [43].

The intake of dietary fluid supplements in the form of functional beverages containing probiotic strains will help with diarrhoea control. The patient could achieve benefit in three ways, as these beverages will be the effective remedial source:

1. Providing lost fluids to the body,
2. Supplementing good gut bacteria to restore gut diversity, which is lost in the condition of diarrhoea (due to any of several reasons as discussed in Section 2),
3. Source of energy to a person suffering from loss of unabsorbed nutrients in frequent bowel movements.

Some of the functional beverages that could be useful in the control of diarrhoea by the provision of above three benefits have been summarised in the following three tables (Table 1, Table 2 and Table 3). 

**Table 1:** Functional dairy-based probiotic beverages to regain gut microbiome lost in diarrhoea.

Functional Food Beverage Products	Probiotic Strains *	Reference
Fermented milk beverage for Normal preparation of Kefir	*Lactococcus lactis*, *Lactobacillus kefiri*	[44]
Fermented milk	Probiotic strains of Lactic acid bacteria and yeasts	[10,45]
Fermented cow’s milk for commercial preparation of Kefir	*Bifidobacterium*, *Lactobacillus acidophilus*, *L. casei*, *L. rhamnosus* and *L. plantarum*	[46]
Fermentation of milk by method of domestic preparation of Kefir	Bacterial strains: *Lentilactobacillus kefiri*, *Leuconostoc mesenteroides* and *Lactococcus lactis* Yeast strains: *Kluvyeromyces marxianus* and *Saccharomyces cerevisiae*	[47]
Fermentation of milk by a method of Industrial preparation of Kefir	Bacterial strains: *Streptococcus thermophilus*, *Lactobacillus delbrueckii* subsp. *bulgaricus* and *Lacticaseibacillus rhamnosus* Yeast strains: *Debaryomyces hansenii* and *Kluvyeromyces marxianus*	[47]
Fermented milk mixed with 1% anthocyanin extracted from black carrots	*Lactobacillus kefiri* (17%), *Leuconostoc mesenteroides* (9%), *Lactococcus lactis* (5%)	[44]
Fermented milk mixed with 5% anthocyanin extracted from black carrots	*Lactobacillus kefiri* (72%), *Streptococcus salivarius* subsp. *thermophilus* (3%)	[44]
Probiotic beverage from Fermented solution of sugar	Various lactic acid bacteria and yeast	[48]
Probiotic beverage from water based medium	*Bifidobacterium tibiigranuli* sp. *nov*.	[49]

* Cultures’ names presented in the table are the same as in corresponding cited references. New nomenclature of bacteria available on: https://isappscience.org/new-names-for-important-probiotic-lactobacillus-species/ (accessed on 15 July 2023). A few examples include: *Lactobacillus casei* as *Lacticaseibacillus casei*; *Lactobacillus reuteri* as *Limosilactobacillus reuteri*; *Lactobacillus rhamnosus* as *Lacticaseibacillus rhamnosus*.

Patients suffering from diarrhoea with lactose intolerance and allergic to milk products or vegan patients can select nutritious dairy-free beverages. These could be prepared from juices extracted from seasonal and locally available fruits and vegetables, some have been summarised in Table 2.

**Table 2:** Functional non-dairy fruit and vegetable-based probiotic beverages to regain gut microbiome lost in diarrhoea.

Functional Food Beverage Products	Probiotic Strains *	Reference
Fruit juice-based probiotic beverages from Pomegranate and Orange	Various lactic acid bacteria and probiotic yeast sourced from a milk-fermented product Kefir	[50]
Fruit juice beverage prepared from Cornelian cherry (*Cornus mas* L.)	Lactic acid bacteria isolated from Kefir grains	[51]
Juices extracted from Guava fruit	*Lactobacillus rhamnosus* ATCC 7469	[52]
Beverage prepared from Pomegranate juice	Cultures isolated from a domestic fermented Kefir	[53]
Mature Coconut water functional fermented beverage	*Lactobacillus plantarum* DW12	[54]
Functional symbiotic Pomegranate beverage	*Lactobacillus paracasei* K5 isolated from Greek Feta-type cheese	[55]
Probiotic beverage from Mediterranean fruit juices from Quince, Grape, Kiwifruit, Prickly Pear and Pomegranate	Various lactic acid bacteria and probiotic yeast sourced from water Kefir	[56]
Probiotic non-fermented blend beverage with Banana, Strawberry and Juçara or Palmiteiro fruit (*Euterpe edulis* Martius)	*Lactobacillus plantarum*, *L. casei*, *Bifidobacterium animalis* subsp. *lactis*	[57]
Functional fermented juice of a mixture of Pineapple, Spinach, Cucumber, Pumpkin and Jerusalem artichoke juices	*Lacticaseibacillus rhamnosus, Lacticaseibacillus paracasei* subsp. *paracasei*, *Lactobacillus acidophilus, Bifidobacterium animalis* subsp. *Lactis* and *Lactiplantibacillus plantarum*	[58]
Cashew-Apple juice, a functional beverage with sweet aroma and reduced astringency	*Lactobacillus plantarum*	[59]
Mixed Pineapple (*Ananas comosus* L. Merril) and Jussara (Euterpe edulis Martius) beverage	*Lactobacillus rhamnosus* GG	[60]
Passion fruit juice in a functional non-dairy product for probiotic delivery	Micro-encapsulated *Bifidobacteria*	[61]
Prickly Pears (*Opuntia* sp.) juice	*Lactobacillus fermentum*—ATCC 9338	[62]
Cherimoya (*Annona cherimola* Mill.) fermented juice matrix for the formulation of stable functional beverages	*Lactobacillus brevis*, *L. plantarum*, *L. rhamnosus* and *Fructobacillus tropaeoli*	[63]
Passion fruit juice	*Lactiplantibacillus plantarum*	[64]
Probiotic mixed Fruit Juices beverage (Apple cider, Orange, Grapes)	*Lactobacillus rhamnosus* GR-1	[65]
Juice from Cantaloupe melon and Cashew fruit (*Anacardium occidentale* L.)	*Lactobacillus casei* NRRL B-442	[66]
Beet and Orange mixed juices (1:1 and 1:2 *v/v*) with 28 days shelf life	*Lactobacillus acidophilus*	[67]
Fruit juice from Apple, Orange and Grapes	*Lactobacillus plantarum*, *L. brevis*, *L. paracasei*, *L. fermentum* and *L. pentosus*	[68]
Fruit juice extracted from Sweet oranges*—Citrus sinensis*	*Lactobacillus casei* (commercial lyophilised culture)	[69]
Encapsulated Probiotic culture added into a matrix of fruits	*L. salivarius* spp. *salivarius*	[70]
Guichang (Kiwi fruit (*Actinidia Lindl*. spp.)	*L. plantarum*	[71]

* Cultures’ names presented in the table are same as in corresponding cited references. New nomenclature of bacteria are available at https://isappscience.org/new-names-for-important-probiotic-lactobacillus-species/ (accessed on 15 July 2023). A few examples include: *Lactobacillus casei* now known as *Lacticaseibacillus casei*; *Lactobacillus reuteri* now known as *Limosilactobacillus reuteri; Lactobacillus rhamnosus* now known as *Lacticaseibacillus rhamnosus***.**

Vegans or patients with lactose intolerance and allergic to milk products can select nutritious beverages prepared from regionally available cereals, grains, beans, nuts, etc. Some of the products have been summarised in Table 3.

**Table 3:** Functional non-dairy cereal-based probiotic beverages to regain gut microbiome lost in diarrhoea.

Functional Food Beverage Products	Probiotic Strains *	Reference
Rice-based Probiotic Beverages	*Limosilactobacillus fermentum* MG7011	[72]
Synbiotic fermented beverage from Soya milk of vegetable soya beans	*Lactobacillus acidophilus* La-5, *Bifidobacterium animalis* Bb-12 in co-culture with *Streptococcus thermophilus*	[73]
Cereal-based probiotic beverages	Lactic acid bacteria strains	[74]
Rice-based fermented beverage	*Lactobacillus fermentum* KKL1	[75]
Soya-based fermented beverage	*Lactiplantibacillus plantarum* CIDCA 8327, *Lacticaseibacillus paracasei* BGP1	[76]
Oat, Coconut cream, Rice flour, based non-dairy-based probiotic beverage	Live vegan kefir cultures *Bifidobacterium*, *Lactobacillus acidophilus*, *L. bulgaricus*, *L. rhamnosus*	[46]
Non-dairy-based Probiotic beverage from Soya milk	Various lactic acid bacteria and probiotic yeast	[77]
Peanut and Soya bean water-soluble extracts	*Lactobacillus rhamnosus* GG	[78]
Maize blended Rice beverages	Commercial probiotic strains *Lactobacillus acidophilus* LACA 4, *Lactobacillus plantarum* CCMA 0743, *Torulaspora delbrueckii* CCMA 0235	[79]
Peanut-Soya milk functional beverage	Binary culture of *Pediococcus acidilactici* and *L. acidophilus*; co-culture of *P. acidilactici*, *L. acidophilus*, *S. cerevisiae*	[80]
Functional Cassava fermented beverage	*Lactobacillus fermentum* CCMA 0215 with yeast strains *Torulaspora delbrueckii* CCMA 0234,0235, *Pichia caribbica* CCMA 0198, *Saccharomyces cerevisiae* CCMA 0232, 0233	[81]
Cassava (*Manihot esculenta* Crantz) and Rice-based beverage with functional properties	*Lactobacillus plantarum* CCMA 0743 (from Cauim), *Torulaspora delbrueckii* CCMA 0235 (from Tarubá) and the commercial probiotic *L. acidophilus* LAC-04	[82]
Blend of Almonds, Oats and Sunflower seeds, a vegan probiotic drink	co-culture LAB + probiotic Yeasts *Lactiplantibacillus plantarum, Pichia kluyveri, P. guilliermondii, Debaryomyces hansenii*	[83]
Cocultured functional probiotic beverage Okara (Soya bean residue) with enhanced nutritional and aroma profiles	*Bifidobacterium lactis*, *Lactobacillus helveticus*, *Lactobacillus paracasei*, yeast *Lindnera saturnus* (*Williopsis saturnus* var. *saturnus*)	[84]
Water-soluble extract of Baru Almond with prebiotics inulin, oligofructose and polydextrose	*Lacticaseibacillus casei*	[85]
Rice-based fermented beverage	*Lactobacillus plantarum* L7	[86]
Quinoa based beverage	*Lactobacillus plantarum* DSM 9843	[87]
Soya and Rice-based drinks	*Lactobacillus fermentum*, *L. plantarum*, *L. helveticus*, *Bifidobacterium bifidum* and *B. longum*	[88]
*Amaranthus hypochondriacus* L. seeds a pseudocereal	*Lactiplantibacillus plantarum*, *Lacticaseibacillus casei* Shirota (as control)	[89]

* Cultures’ names presented in the table are same as in corresponding cited references. New nomenclature of bacteria are available on https://isappscience.org/new-names-for-important-probiotic-lactobacillus-species/ (accessed on 15 July 2023). A few examples include: *Lactobacillus casei* now known as *Lacticaseibacillus casei*; *Lactobacillus reuteri* now known as *Limosilactobacillus reuteri; Lactobacillus rhamnosus* now known as *Lacticaseibacillus rhamnosus*.

## 5. Conclusions and Emerging Trends in Nutraceutical Beverages

Although most cases of diarrhoea do not require medical therapy, it is still very important for a person suffering from frequent bouts of watery bowel movements, that might be causing substantial loss of fluid from the patient’s body, to avoid the condition of dehydration. Drinks prepared using salts and sugars in water are normally given to patients for the recovery of hydration and nutrients lost in the condition of diarrhoea. However, healthier drinks in the form of probiotic beverages could be combinational therapy options for such patients. These nutraceutical beverages could be prepared with nutritious dietary materials like seasonal fruits, vegetables and other agricultural resources like grains, cereals, beans, nuts, etc. The organoleptic properties of fermented beverages could be enhanced with the addition of ingredients from edible plant sources for flavours and required antimicrobial bioactivities for the control of gastric infection.

The prospects and emerging trends of bioactive molecules extracted from natural resources including culinary herbs, dietary items like fruits and vegetables, extracts from medicinal plants, etc. have been identified for their possible application as therapeutic agents, that can be used for fortification of beverages for several health benefits including gut infections and disorders in the digestive system [90,91]. The bioactive compounds with antimicrobial and other therapeutic activities have been investigated in plant-sourced edible materials [92,93,94,95,96]. Products prepared using such bioactive materials are free from chemically synthesised molecules, for instance, commonly prescribed antibiotics have chemically-synthesised compounds. Hence, naturally occurring materials with pharmaceutical importance [97,98,99,100] can be combined with the activities of characterised probiotic cultures in fermented beverages or in a formulation for supplementation. The intake of either option according to the choice of a patient can help in regaining friendly gut microbiota lost during the period of diarrhoea. There is always a scope for further research on the improvement of probiotic beverages with nutraceutical therapeutic applications. Emerging trends should be aimed at using a strategy of chemical-free, natural, safe and economical therapy for controlling diarrhoea, gut infections and diseases related to any part of the gastrointestinal tract.

## Data Availability

Not applicable.
